# Neoadjuvant Therapy of Pancreatic Cancer: Definitions and Benefits

**DOI:** 10.3390/ijms18081622

**Published:** 2017-07-26

**Authors:** Stefan Heinrich, Hauke Lang

**Affiliations:** Department of General, Visceral and Transplantation Surgery, University Hospital of Mainz, Langenbeckstrasse 1, 55131 Mainz, Germany; hauke.lang@unimedizin-mainz.de

**Keywords:** pancreatic cancer, neoadjuvant therapy, chemotherapy, chemoradiation therapy, borderline resectable

## Abstract

The standard treatment of resectable pancreatic cancer is surgery followed by adjuvant chemotherapy. Due to the complication rate of pancreatic surgery and the high rate of primary irresectability, neoadjuvant concepts are increasingly used for pancreatic cancer. Neoadjuvant therapy is better tolerated than adjuvant and might decrease the surgical complication rate from pancreatic surgery. In contrast to neoadjuvant chemoradiation, the nutritional status improves during neoadjuvant chemotherapy. Also, the survival of patients who develop postoperative complications after neoadjuvant therapy is comparable to those without complications whereas the survival of patients who underwent upfront surgery and then develop surgical complications is impaired. Moreover, large data base analyses suggest a down-sizing effect and improvement of overall survival by neoadjuvant therapy. Neoadjuvant chemotherapy appears to be equally efficient in converting irresectable in resectable disease and more efficient with regard to systemic tumor progression and overall survival compared to neoadjuvant chemoradiation therapy. Despite these convincing findings from mostly small phase II trials, neoadjuvant therapy has not yet proven superiority over upfront surgery in randomized trials.

## 1. Introduction

The standard treatment for patients with resectable pancreatic cancer is surgery followed by adjuvant chemotherapy (CTx): after the ESPAC-1 trial had suggested a survival benefit for an adjuvant CTx, Oettle et al. have demonstrated a significant improvement of the recurrence-free and overall survivals for patients with pancreatic cancer after a curative resection. Further trials have confirmed these results and have shown survival improvements for combination CTx [1]. Patients with metastatic or unresectable pancreatic cancer are usually offered palliative therapy. Depending on the symptoms and the general condition of the patient, chemoradiation therapy (CRTx) or systemic CTx with gemcitabine, nab-Paclitaxel or Folfirinox have become standard of care [2,3].

Theoretical advantages of neoadjuvant over adjuvant treatments are the treatment of circulating tumor cells and micrometastases prior to surgery, higher probability of R0 resection rates and a better patient selection due to exclusion of patients from surgery with progressive disease under neoadjuvant treatment. On the other hand, toxic neoadjuvant regimen may be harmful since these would hamper the surgical outcome. On the other hand, some (surgeons) may consider the risk of disease progression under therapy as a major disadvantage for the patient.

Neoadjuvant therapy has become the standard of care for most gastrointestinal cancers: randomized trials have demonstrated beneficial effects for esophageal, gastric and rectal cancer [4,5,6]. In contrast to general concerns, these studies have demonstrated that neoadjuvant is better tolerated than adjuvant therapy, and that patients receive more treatment, if it is started preoperatively [4,5].

Considering these findings from other gastrointestinal cancers, the introduction of neoadjuvant therapy for pancreatic cancer appears reasonable and has gained massive popularity over the past 20 years [7]. While initial reports from a few US centers only reported on neoadjuvant CRTx, the majority of current publications reports on neoadjuvant CTx. This trend has become overt since the introduction of Folfirinox and nab-Paclitaxel/gemcitabine regimen into the palliative treatment of pancreatic cancer.

Although several analyses suggest beneficial effects of neoadjuvant therapy for pancreatic cancer, no randomized controlled trials have yet proven superiority of neoadjuvant therapy over upfront surgery.

The basis of this review is an extensive PubMed literature research on the latest publications on the respective fields. Whenever available, review articles were accepted if the methodology was felt appropriate for the question of this review.

## 2. Definition of Resectability

Due to the close contact to the superior mesenteric vessels and the celiac arteries, resectability of localized pancreatic cancer is often hampered by the infiltration of these structures. Since vascular resections are considered critical or even impossible by many surgeons, the resectability of such advanced tumors is highly depending on the individual expertise of the surgeon.

In contrast to single-center series, recent meta-analyses have demonstrated that venous and arterial resections are associated with a higher perioperative mortality and impaired long-term survival [8]. Thus, the long-term outcome after resection of the superior mesenteric vein (SMV) largely depends on the depth of infiltration of the venous wall suggesting that many patients with limited SMV infiltration have an unaffected long-term survival [9]. Moreover, an arterial resection may be the only chance of a local tumor clearance, and therefore, the concept of arterial resections is increasingly used for cancer of the pancreatic body (“Appleby operation”) or in locally advanced cancer after neoadjuvant treatment. For these reasons, patients with arterial or venous tumor involvement are nowadays also considered surgical candidates under certain instances.

**Figure 1:**
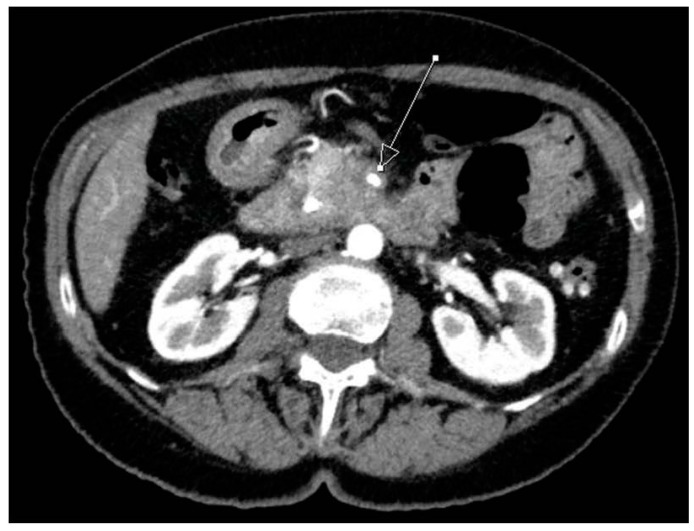
Computed tomography image of a tumor in the pancreatic head which surrounds the superior mesenteric artery (arrow: “unresectable”).

Considering these continuous changes in the surgical attitude, it is no wonder that resectability criteria varied among surgeons and associations. The current concept of local resectability is based on the extent of vascular involvement: tumors without contact to the major mesenteric or celiac vessels are considered resectable. In contrast, a tumor contact to the superior mesenteric or hepatic arteries of more than 180° is currently considered unresectable as well as an infiltration of the superior mesenteric vein (SMV) without the possibility of reconstruction (Figure 1). The current classifications of (un-)resectability are largely uniform, although the extent of venous involvement and the infiltration of the celiac and hepatic arteries varies [10,11,12,13].

**Figure 2:**
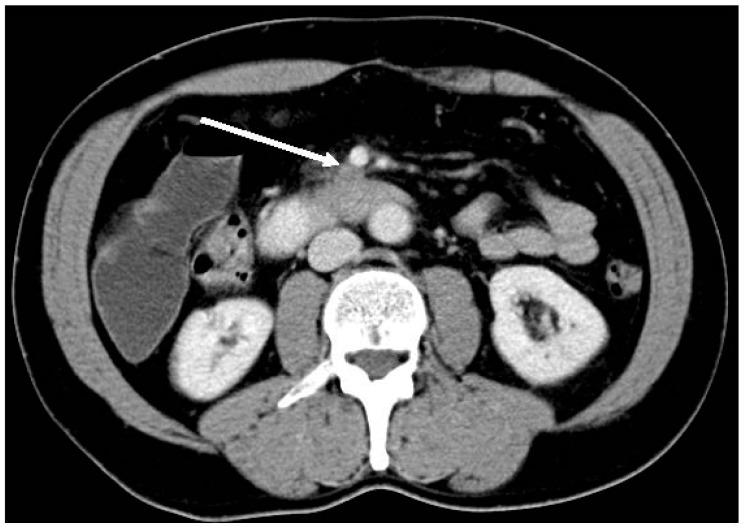
Computed tomography image of a tumor in the uncinate process with tumor contact to the superior mesenteric vein (smv) over a distance of about 2 cm (arrow: “borderline resectable”).

Those tumors which are neither classified as resectable nor unresectable are classified as borderline resectable (Figure 2). While some surgeons will consider such borderline resectable tumors for surgery, others would deny these patients surgery depending on their expertise and surgical attitude. Despite different approaches to borderline resectable tumors, a uniform definition of resectability would be helpful for the comparability of clinical studies [10,11,12,13].

Of note, the concept of borderline resectability also introduces major bias in several ways: the spectrum of “borderline” includes a wide range of clinical situations: a short-segment infiltration of the SMV close to the confluence has a higher chance of R0 resection than a tumor contact to the superior mesenteric artery (SMA) up to 180°—both are classified as borderline resectable. Moreover, secondary resectability is a difficult primary study endpoint of clinical studies on neoadjuvant therapy for borderline resectable pancreatic cancer considering the different surgical attitudes to the treatment of this subgroup. Therefore, the definitions of primary and secondary (ir-)resectability must be analyzed with caution when comparing these end-points of different studies after neoadjuvant therapy.

Further terms used in the literature are “potentially resectable” and “locally advanced” Both terms do not have a clear definition. While potentially resectable summarizes resectable and borderline resectable disease, locally advanced is generally considered unresectable disease without distant metastases at this moment (Table 1) [14]. Unresectability used to trigger palliative treatments in the past, since cure was considered impossible. However, secondary resectability can also be achieved in such advanced tumors with the modern more powerful (radio-)CTx regimen.

## 3. Definition of Neoadjuvant Therapy

The aim of an adjuvant therapy is the reduction of tumor recurrence after an apparently curative resection, and the aim of a palliative therapy is the relief of symptoms in incurable disease. Both are usually associated with a survival benefit, which, however, might be minimal or absent for palliative therapy. While adjuvant and palliative therapies are well defined, a clear definition of “neoadjuvant” therapy is currently lacking. Any preoperative therapy in resectable cancer as well as therapies which might result in surgery in case of tumor response, are considered “neoadjuvant” in the literature. Furthermore, a pretreatment of patients with synchronously metastatic disease (e.g., liver metastases) is also considered “neoadjuvant”, if a resection of the pancreatic primary and the liver metastases may be considered in a subset of patients without tumor progression. Consequently, the current literature varies widely regarding the patient populations for neoadjuvant therapy.

Neoadjuvant therapy may be based on different treatment modalities. Due to the lack of an efficient chemotherapy for pancreatic cancer, neoadjuvant therapy was historically based on CRTx [15] Gemcitabine mono-therapy revealed some clinical benefits in the palliative treatment, but is basically inefficient in the neoadjuvant setting [16]. Gemcitabine-based combination chemotherapy showed superiority over gemcitabine-mono therapy. Such combinations were first tested in resectable pancreatic cancer due to the acceptable toxicity [17]. With the developments of polychemotherapy, neoadjuvant chemotherapy has become more attractive. While CRTx offers the advantage of a high-dose local treatment, chemotherapy offers the benefit of a systemic treatment of micrometastases. Due to the high response rates of Folfirinox and nab-Paclitaxel in the palliative situation, these protocols have gained increasing interest in particular for borderline and unresectable cancer during recent years [2,3].

## 4. Rationale for Neoadjuvant Therapy

It appears reasonable to treat patients with locally advanced tumors with the aim of achieving secondary resectability (downsizing), since surgery is considered the only curative treatment option for these patients. Consequently, most of the literature on neoadjuvant treatment is about patients with borderline or locally advanced (unresectable) pancreatic cancer. Due to the high local effect, CRTx has historically been preferred in this situation. However, a recent analysis of 575 patients with locally advanced pancreatic cancer revealed higher secondary resectability rates after Folfirinox chemotherapy compared to gemcitabine-based CRTx [18].

Another, oncologically much more interesting concept is the preoperative treatment of patients with primarily resectable pancreatic cancer to decrease recurrence rate and to improve long-term survival [16].

### 4.1. Effects of Surgical Complications on Multimodality Treatment

Perioperative mortality has dramatically improved during the past 30 years, and large database analyses have demonstrated lower perioperative mortality and better long-term survival in large-volume centers [19,20]. However, the procedure-related morbidity remains high with pancreatic fistula (~20%) being the most important complication [21].

Due to the substantial complication rate and negative impact on the quality of life related to pancreatic surgery, about 40% of patients deny further treatments or are not considered eligible for adjuvant therapy by their treating physicians [22]. Consequently, patients who suffer surgical complications do not benefit the effect of an adjuvant treatment and have an impaired overall survival [23].

Tzeng et al. analyzed the MD Anderson Cancer Center database (2002–2007) for the effect of surgical complications on the outcome of multimodality therapy. The majority of patients (*n* = 115) received neoadjuvant therapy, while only 50 patients were operated upfront with potential adjuvant therapy. In this analysis, the survival of patients who completed a multimodality treatment was significantly superior to those who did not. These patients with complications from surgery had the same survival as patients who underwent neoadjuvant therapy without surgery. In contrast, survival did not differ between patients with or without major surgical complications, if they had received neoadjuvant therapy. Moreover, the long-term survival of patients who completed adjuvant therapy without surgical complications was not different from patients after neoadjuvant therapy [24].

In the O’Reilly et al. study on neoadjuvant CTx for resectable pancreatic cancer, 26/27 (96%) patients received adjuvant therapy, and in the Sho et al. study on neoadjuvant CRTx, 95% of the patients (compared to 90% after upfront surgery) also received adjuvant CTx after neoadjuvant CRTx [25,26].

### 4.2. Effects of Multimodality Treatment on the Surgical Complication Rate

A preoperative therapy is generally well tolerated, and such therapies do not complicate surgery, if the regimen is not too toxic. In phase II trials on neoadjuvant CTx, no patient denied surgery due to toxicity of the neoadjuvant therapy [26,27]. Interestingly, the surgical complication rate was very low after neoadjuvant CTx in phase II trials: in particular, the incidence of pancreatic fistula was extremely low in several studies on neoadjuvant chemo- or CRT therapy [28]. A recent meta-analysis confirmed thee low surgical complication rates after neoadjuvant CTx and CRTx, and this complication rate appeared to be lower than reported morbidity for surgery-only patients in the literature [28].

Several explanations are possible for this observation: neoadjuvant CTx improves the nutritional status and by this may decrease the surgical complication rate (see below). Furthermore, neoadjuvant therapy may induce pancreatic fibrosis, which would result in a lower incidence of pancreatic fistula.

### 4.3. Effect of Neoadjuvant Therapy on Nutritional Status

Malnourished patients have a higher perioperative complication rate than patients with a normal nutritional status. Malignant diseases in general are associated with an impaired nutritional status due to unknown mechanisms [29]. In addition, biliary obstruction (e.g., cancer of the pancreatic head) causes malnutrition due to the lack of intestinal bile, which is reversed by the restoration of bile flow [30,31]. A general concern against neoadjuvant therapy is the potentially detrimental effect on the general condition of the patient due to treatment-associated toxicity.

Indeed, Naumann et al. have demonstrated that the patients’ nutritional status indeed deteriorated during neoadjuvant CRTx in a phase II trial, in which body weight and skin fold thickness were used as measures of the nutritional status [32]. In this study, the deterioration of the nutritional status was most prominent in patients with grade III emesis. A similar observation was reported by Sho et al. from a phase II trial on neoadjuvant CRTx for resectable pancreatic cancer [25].

In contrast, only very few patients developed relevant emesis after neoadjuvant CTx with gemcitabine/cisplatin. In this study, 42% of the patients had abnormal prealbumin serum levels at study entry, and the prealbumin serum levels significantly improved during the neoadjuvant treatment. At surgery, only three patients had abnormal prealbumin serum levels. This observation may be attributed to a restoration of bile flow by stent placement and two months of chemotherapy with low toxicity profile [27].

### 4.4. Histological Response

The extent of the treatment effect of a neoadjuvant therapy can be objectively scored by the pathologist after a resection of the primary tumor.

Evans et al. published a score for the assessment of the histological response to neoadjuvant therapy, which is based on the proportion of tumor necrosis in response to the treatment. In their initial report, 28 patients were treated by CRTx, of which 17 underwent tumor resection. Out of these, histological tumor response was graded II in 11 patients (65%) and III in 4/17 patients (24%) (Table 2) [15].

When this score was adopted to assess the histological response to neoadjuvant CTx, 13 patients (54%) had II, but higher degrees of histological response were not observed. Moreover, more than 80% of the tumors revealed cytopathic effects in response to the neoadjuvant treatment [27]. In the same study, low standardized uptake values (SUV) in positron-emission-tomography scans at study entry correlated significantly with the degree of morphological response [37].

In an analysis of nearly 15,000 patients of the US National Cancer Database from 2006 to 2012, tumors after neoadjuvant therapy had lower T- and N-stages. Moreover more patients had R0 resections after neoadjuvant therapy [38]. Assuming that currently patients with more advanced local tumor stages are preferably considered for neoadjuvant therapy, these results confirm the data on histological response from phase II trials.

A recent meta-analysis of the current literature confirms these findings of lower T- and N-stages, lower rates of perineural and lymphovascular infiltration rates as well as higher R0-resection rates after neoadjuvant therapy [39].

### 4.5. Response Prediction

The standard (re-)staging examination for pancreatic cancer is computed tomography (CT), which depicts vascular invasion and distant metastases. After neoadjuvant therapy, new metastases may be detected by CT-scanning. However, the exact local tumor regression in response to neoadjuvant therapy is not exactly described by CT [40]. Therefore, surgical exploration is recommended by most authors to evaluate local resectability in primarily borderline or unresectable tumors [40]. As for other types of cancer, an early prediction of the tumor response to a neoadjuvant therapy would be highly appreciated particularly for primarily resectable disease [41,42]. Such an early response prediction could exclude patients from a neoadjuvant therapy in case of an inefficient therapy.

#### 4.5.1. Response Prediction by CA19.9 Serum Levels

Most cancers of the pancreatico-biliary tract express the tumor marker CA 19.9, and this serum marker is therefore considered the standard tumor marker for pancreatic cancer. In the palliative treatment of patients with pancreatic cancer CA 19.9 is a well-established response marker [43]. Also, CA 19.9 appears to be prognostic in early stage pancreatic cancer [44]. Due to the high rate of false positive values in cholestatic patients, CA 19.9 serum levels are however not diagnostic in resectable pancreatic cancer.

In the setting of neoadjuvant therapy, CA 19.9 serum levels have also been investigated in several phase II trials. In most studies, CA 19.9 serum levels decreased during the neoadjuvant therapy. Although low CA 19.9 levels after neoadjuvant therapy were associated with higher resectability rates, a decrease in CA 19.9 serum levels in response to therapy was not associated with the radiological [45], histological response or overall survival [37,46,47,48,49,50,51]. Although the utility of CA 19.9 was judged very limited in their initial paper, Tzeng et al. also showed that a complete normalization of CA 19.9 levels was associated with improved overall survival in a follow-up paper [51]. A similar effect of a >50% decrease in CA 19.9 serum levels on overall survival was reported by Boone et al. [52].

The lack of significance of the CA 19.9 response in the setting of neoadjuvant therapy in the majority of studies and the controversies between these studies is mainly due to various time points of the pre-therapeutic CA 19.9 measurements and the heterogeneity of the treatments. Mellon et al. only used CA 19.9 levels after biliary stenting, and Katz et al. and Tzeng only included CA 19.9 levels in the absence of hyperbilirubinemia [46,49,51]. In these studies, the degree of CA 19.9 response was not associated with the degree of histological response, although Mellon et al. revealed lower CA 19.9 levels in partial and complete histological response.

None of the published studies evaluated CA 19.9 serum levels during the treatment. Considering this limitation and the controversial results of the literature, serum CA 19.9 levels are not suitable for early prediction of the histological response to neoadjuvant therapy.

#### 4.5.2. Response Prediction by Positron-Emission-Tomography (PET)

PET based on ^18^Fluoro-Desoxy-Glucose (FDG) has been used for several tumor entities to predict tumor response to neoadjuvant therapy [41,42]. For esophageal cancer PET scanning has even be proposed to select patients very early during neoadjuvant therapy, since only responders benefit from this treatment [53].

For pancreatic cancer, PET-scanning has also been used pre- and post-therapeutically, and FDG-uptake decreased during neoadjuvant therapy in all studies [37,45,54,55]. Low post-treatment FDG-uptake did not correlate with radiological [41], but was associated with higher histological responses [37,55]. However, PET-scanning after two or four weeks of therapy in order to predict tumor response early has not been investigated in neoadjuvant therapy for pancreatic cancer, yet.

### 4.6. Disease Progression under Neoadjuvant Therapy

Since the assessment of borderline resectable and locally advanced disease varies between centers depending on the surgical expertise, a potential tumor progression under neoadjuvant therapy, which precludes surgery, can only be evaluated in primarily resectable pancreatic cancer.

The resectability rate after neoadjuvant CTx for resectable pancreatic cancer was 77% in the O´Reilly trial and 89% in the study reported by Heinrich et al. [26,27]. In both trials, laparoscopy was performed before neoadjuvant therapy in order to exclude peritoneal carcinomatosis or superficial liver metastases. In the study of Heinrich et al. two patients had peritoneal carcinomatosis after neoadjuvant therapy, of which one was included without diagnostic laparoscopy prior to chemotherapy, and one patient required cardiac prior to pancreas surgery, which resulted in a significant treatment delay. In the O’Reilly study, three patients had metastases found during exploration for pancreas surgery. Moreover, both trials report locally unresectable disease in one and five patients respectively [26,27]. A randomized phase II trial has demonstrated that combination chemotherapy (gemcitabine/cisplatin) is more efficient than gemcitabine-mono therapy [16].

Despite the higher local effect of neoadjuvant CRT as measured by the histological response, the resectability rates after neoadjuvant CRTx appear lower with 58–74% compared to neoadjuvant CTx [15,34,35]. This is most probably due to the higher rate of systemic tumor progression in the phase II trials on neoadjuvant CRTx due to the lack of systemic effect of the CTx element (Table 2).

### 4.7. Effect on Survival

Due to the lack of adequately powered randomized trials, the current evidence for an effect of neoadjuvant therapy on long-term outcome arises from phase II trials and retrospective database analyses. An extensive review of the literature regarding the survival estimates of neoadjuvant treatments has been performed by Gillen et al. [56]. According to this review, the overall survival of patients undergoing pancreas resection after neoadjuvant therapy is slightly better than the survival of those who receive adjuvant therapy after a potentially curative pancreas resection. According to this meta-analysis, patient survival after pancreas resection without adjuvant therapy appeared worst (16.9–20.2 months) compared to patients who did receive adjuvant therapy (20.1–23.6 months). Patients with resectable cancer who received neoadjuvant therapy revealed an overall survival of 23.3 months, and even patients with locally unresectable cancer revealed an overall survival of 20.5 months if a resection was performed after successful neoadjuvant therapy [56].

Neoadjuvant chemotherapy for primarily resectable disease has only been reported from a few centers. These phase II trials report a median survival of 26.5 months and 27.2 months after two-months of neoadjuvant CTx with or without adjuvant CTx [26,27]. Most of the patients in these studies had T3 and N1 cancers. Moreover, all patients, irrespective of surgical complications, were included into these trials in contrast to the randomized trials on adjuvant CTx, in which only patients were included who recovered well from surgery without major complications. Despite this positive patient selection, survival in these randomized trials was not better than in the phase II trials on short-term neoadjuvant therapy.

Very recent large database analyses also suggest a survival benefit for patients who received neoadjuvant therapy [38]. Interestingly, most of these patients received neoadjuvant CTx without radiation therapy. According to two separate analyses of the national cancer database, perioperative chemotherapy is associated with a survival benefit in patients with early stages (I and II) pancreatic cancer over upfront surgery. Moreover, neoadjuvant CTx appears more efficient than CRTx in early stage pancreatic cancer [57,58]. However, the inclusion criteria and the local extent of the disease are not clearly stated in these analyses.

## 5. Discussion

The current standard treatment for patients with pancreatic cancer is surgery followed by adjuvant CTx. However, a significant proportion of patients cannot undergo surgery or receive adjuvant therapy [24]. Many patients present with locally unresectable disease or with disease at a high risk of incomplete resection. Considering most recent data on the survival advantage of larger resection margins (R0 vs R1_1mm_ vs. R1_direct_), both subgroups of patients should be candidates for neoadjuvant therapy [59]. Moreover, pancreas surgery inherits a significant morbidity and extended resections for advanced tumors have an even higher perioperative morbidity, which is a major reason for not receiving adjuvant treatment [24]. Consequently, these patients are undertreated regarding the tumor disease.

Currently, neoadjuvant therapy is mainly used for borderline and unresectable pancreatic cancer, when surgery is considered inefficient or impossible. Due to its stronger local efficacy, CRTx has been preferred over chemotherapy to down-size the tumor and enable secondary resectability for a long time (Table 2).

Since neoadjuvant is generally better tolerated than adjuvant therapy, patients are most likely to receive a sufficient treatment dosage and have the highest probability to receive a multimodality treatment at all, if the treatment starts prior to surgery—also for primarily resectable cancer. Several phase II trials and large database analyses suggest beneficial effects of neoadjuvant therapy including low surgical complication rate, high treatment exposure and local treatment effect with convincing overall survival. Although one might argue that patients with resectable pancreatic cancer should not be put at risk of treatment related complications or disease progression due to an ineffective treatment, this effect is extremely rare. In contrast, a large proportion of patients who undergo upfront surgery will not benefit the effects of an adjuvant therapy due to surgical complications [24]. Therefore, patients with primarily resectable pancreatic cancer could also be candidates for neoadjuvant therapy, since they would benefit in several aspects: in addition to the treatment of micrometastases and a potential increase of the R0-resection rate, neoadjuvant therapy could decrease the surgical complication rate and potentially improve the nutritional status of the patients. If patients develop surgical complications after neoadjuvant therapy, these patients seem to have a better survival than if they had upfront surgery. Furthermore, patients with an uncomplicated postoperative course will also have a higher probability of receiving adjuvant therapy in addition.

Interestingly, the use of neoadjuvant therapy has expanded over the past years without a clear scientific background [58]. However, given the limited prognosis of patients with pancreatic cancer and the proven advantages of neoadjuvant therapy for most other gastrointestinal cancers, this tendency appears reasonable. Why should a neoadjuvant/perioperative therapy be beneficial for esophageal, gastric or rectal cancer and harmful for pancreatic cancer?

In theory, the rationale for neoadjuvant therapy in patients with resectable pancreatic cancer would be the treatment of micrometastases prior to surgery, while the rationale in patients with unresectable cancer would be the secondary resectability of the tumor. These rationales are merging in patients with borderline resectable disease. Although the definitions of local resectability appear clear in the literature, the different groups of resectability are largely overlapping in the clinical routine. Consequently, the data on borderline resectable and irresectable cancer cannot be separately analyzed. Also, most studies on resectable tumors include borderline resectable tumors since they were judged technically resectable.

The current literature suggests a stronger local effect of neoadjuvant CRTx considering the histological response to the treatment [33,34,35]. Moreover, a recent analysis of Folfirinox-based neoadjuvant CRTx in borderline resectable cancer revealed a median survival of 21.7 months. Also, Rose et al. achieved a 48% resectability rate in borderline resectable pancreatic cancer by Gem/doxitoxel followed by radiotherapy in case of little response to chemotherapy [60]. On the other hand, small randomized trials have not shown a survival benefit for neoadjuvant CRTx [35,36], which is probably due to the higher rate of systemic tumor progression. In contrast, neoadjuvant CTx appears to have a lower toxicity, and seems to result in a better overall survival (Table 2). Neoadjuvant Folfirinox resulted in about 25% secondary resectability and a median survival of 24.2 months in a recent meta-analysis, making this chemotherapy a valuable tool for locally advanced disease [61].

Moreover, the estimated probability of micrometastases at the moment of surgery for most patients with pancreatic cancer is very high [62]. Since systemic chemotherapy exhibits a significant local tumor response and is effective for the treatment of systemic disease (micrometastases), a neoadjuvant (systemic) CTx appears most reasonable for primarily resectable tumors. This reading of the literature is supported by the analyses of Lutfi et al., which show that neoadjuvant CTx resulted in a survival benefit in early-stage pancreatic cancer patients, whereas CRTx was not superior to chemotherapy alone [57]. The stronger local effect of CRTx would theoretically be most effective in locally unresectable tumors due to the stronger down-sizing effect. But, a recent comparative analysis revealed a higher secondary resectability rate after neoadjuvant Folfirinox over gemcitabine-based CRTx [18].

Alternatively to neoadjuvant CTx and CRTx for locally advanced pancreatic cancer, local ablative treatments such as irreversible electroporation (IRE) and high intensity focused ultrasound (HIFU) have been introduced into the management of locally unresectable tumors [63,64,65]. These technologies appear very attractive due to the low treatment associated morbidity. However, the clinical value of these technologies is still unclear, in particular since they are frequently part of multimodal treatment protocols.

The interpretation of the current literature is still difficult due to differences in definitions and inclusion criteria. First, different definitions for “neoadjuvant” are used in the literature. Also, the definition of local resectability varies between the studies and is highly dependent on the individual expertise of the center. Also, the concept of borderline resectability is not followed in the recent trials on neoadjuvant therapy (Table 1). Moreover, different CRTx regimen have been published making the interpretation of the current literature on CRTx and the comparison of the oncological efficacy to chemotherapy extremely difficult. What can be extracted from the current literature is, that the general concerns against neoadjuvant therapy are not justified. The current literature demonstrates plenty of advantages of neoadjuvant therapy, which require confirmation from large randomized trials.

Unfortunately, solid randomized trials are lacking in order to confirm the theoretical advantages of neoadjuvant therapy. Major problems of trials on neoadjuvant therapy are the different attitudes of surgeons and oncologists and the preoperative cytological/histological confirmation of adenocarcinoma. Thus, randomized trials are highly anticipated to clarify which patient benefits from neoadjuvant therapy, and to define the ideal multimodality treatment for resectable, borderline and unresectable pancreatic cancer. Although all centers agree that patients with locally advanced disease and those with a high risk of an R1-resection should receive neoadjuvant therapy in order to increase the chance of a complete resection, patients with primarily resectable cancer potentially benefit the most from neoadjuvant therapy. Before new treatment combinations are tested for the different scenarios, such randomized trials should prove the concept of neoadjuvant therapy for the different indications. Following the dramatic improvements in surgical outcome of pancreatic surgery many studies are currently performed in order to test new treatment concepts and to evaluate the true role of neoadjuvant therapy. These studies should be actively supported in order to further improve long-term outcome of patients with resectable, borderline resectable and primarily unresectable pancreatic cancer (f.e. [66,67,68,69]).

## Figures and Tables

**Table 1:** Current concept of resectability (definitions vary slightly between major associations).

	Resectable	Borderline Resectable	Unresectable/Locally Advanced
SMA/CA	No contact	<180°	>180° involvement
PV/SMV	No contact	>180°	No reconstruction possible
	Potentially resectable		

**Table 2:** Summary of studies on neoadjuvant chemo- and chemoradiation therapy for “resectable“ pancreatic cancer.

Author	*n*	Inclusion Criteria		Treatment	Hematologic Toxicity ^1^		GI Toxicity ^1^		Hosp ^2^	Median Survival	Systemic Disease Progression	Evans Score				
					III	IV	III	IV				I	IIa	IIb	III	IV
Evans et al. [33]	86	potentially resectable	(1) no evidence of extrapancreatic disease	Gemcitabine (400 mg/m^2^) Radiation (30 Gy)	69	6	60	0	40	22.7 months	17/86 (19.7%)	12 (19%)	15 (23%)	28 (44%)	8 (13%)	1 (1%)
			(2) no evidence of tumor extension to the (SMA) or celiac axis													
			(3) no evidence of occlusion of the (SMV) or SMV–PV confluence													
			(4) Tumor abutment and encasement of the SMV, in the absence of vessel occlusion or extension to the SMA was considered resectable													
Varadachary et al. [34]	90	resectable	As Evans et al.	gemcitabine (750 mg/m^2^) cisplatin (30 mg/m^2^) radiation (30 Gy) gemcitabine (400 mg/m^2^)	54		28		42	17.4 months	19/90 (21.1%)	2 (11%)	18 (20%)	14 (15.5%)	8 (8.9%)	1 (1.1%)
Sho et al. [25]	61	resectable	(1) no distant metastases	Gemcitabine (1000 mg/m^2^) Radiation (50–54 Gy)	67	5	0	0	-	28 months	0%	1 (2%)	35 (57%)	21 (34%)	20 (32%)	2 (3%)
			(2) tumor abutment with impingement and narrowing of the portal vein lumen													
			(3) encasement of the SMV/portal vein allowing for safe resection and reconstruction													
			(3) no extension to the celiac axis													
			(4) tumor abutment of the SMA not to exceed 180°													
Golcher et al. [35]	33	resectable	Resectability:	Surgery	0	0	0	0		14.4 months						
			(1) no organ infiltration except the duodenum	versus												
	33		(2) maximal involvement of peripancreatic vessels ≤180°	gemcitabine 300 mg/m^2^ cisplatin 30 mg/m^2^ Radiation (50.4 Gy)	18	4	11	0		17.4 months	-	-	-	-	-	-
Casadei et al. [36]	20	resectable	Tumors were resectable:	Surgery						19.5 months						
			(1) no distant metastases	versus												
	18		(2) less than180° involvement of SMV/PV (Ishikawa 0–2)	gemcitabine 1000 mg/m^2^ Radiation (45Gy) gemcitabine 50 mg/m^2^	5	1	0	0		22.4 months	4 (22.2%)	None ^4^	Minimal	Small	Moderate	Large
			(3) clear fat planes around the CA, HA, SMA									0	2 (11.1%)	3 (16.7%)	5 (27.8%)	1 (5.6%)
Heinrich et al. [27]	28	resectable	(1) Patients with infiltration of the superior mesenteric or celiac arteries (T4) were excluded.	Gemcitabine (1000 mg/m^2^) Cisplatin (50 mg/m^2^)	2	0	6	0	-	26.5 months	2/28 (7.1%)	11 (46%)	5 (20%)	8 (34%)	0	0
			(2) Patients with infiltration of the portal vein, the duodenum, or the stomach (T3) were not excluded													
O´Reilly et al. [26]	38	resectable	(1) clear fat plane around celiac and superior mesenteric arteries and a patent superior mesenteric vein and portal vein without primary tumor involvement	Gemcitabine (1000 mg/m^2^) Oxaliplatin (80 mg/m^2^)	0	0	1	0	-	27.2 months	3/35 (8.6%)	-	-	-	-	-
			(2) no encasement of the superior mesenteric vein or portal vein involvement													
			(3) no encasement of the superior mesenteric artery or hepatic artery													
			(4) no extra-regional nodal disease													
Palmer et al. [16]	24 ^3^	resectable	(1) Patients with tumor surrounding >180% of the PV or SMV or	gemcitabine (1000 mg/m^2^)	12		0		0	9.9 months	-	-	-	-	-	-
			(2) direct tumor extension to SMA or CA	versus												
	26		(3) evidence of extra-pancreatic disease were considered nonresectable	gemcitabine (1000 mg/m^2^) cisplatin (25 mg/m^2^)	10		0		6	15.6 months						

^1^ events; ^2^ hospitalization; ^3^ No histological confirmation required for study inclusion (final histology: Gem: *n* = 20 adenocarcinoma, Gem/Cis: *n* = 17 adenocarcinoma); ^4^ according to Rebekah et al.,”-“: not reported, PV: portal vein, SMA: Superior mesenteric artery, CA: Celiac axis (artery). HA: Hepatic atery.
